# Depression-related anterior cingulate prefrontal resting state connectivity normalizes following cognitive behavioral therapy

**DOI:** 10.1192/j.eurpsy.2020.34

**Published:** 2020-04-14

**Authors:** Spiro P. Pantazatos, Ashley Yttredahl, Harry Rubin-Falcone, Ronit Kishon, Maria A. Oquendo, J. John Mann, Jeffrey M. Miller

**Affiliations:** 1 Molecular Imaging and Neuropathology, New York State Psychiatric Institute, New York, New York, USA; 2 Department of Psychiatry, Columbia University Irving Medical Center, New York, New York, USA; 3 Department of Electrical Engineering and Computer Science, University of Michigan, Ann Arbor, Michigan, USA; 4 Department of Psychiatry, Perelman School of Medicine, University of Pennsylvania, Philadelphia, Pennsylvania, USA

**Keywords:** Cognitive behavioral therapy, depression, functional magnetic resonance imaging, resting-state functional connectivity, subgenual cingulate cortex

## Abstract

**Background.:**

Aberrant activity of the subcallosal cingulate (SCC) is a common theme across pharmacologic treatment efficacy prediction studies. The functioning of the SCC in psychotherapeutic interventions is relatively understudied, as are functional differences among SCC subdivisions. We conducted functional connectivity analyses (rsFC) on resting-state functional magnetic resonance imaging (fMRI) data, collected before and after a course of cognitive behavioral therapy (CBT) in patients with major depressive disorder (MDD), using seeds from three SCC subdivisions.

**Methods.:**

Resting-state data were collected from unmedicated patients with current MDD (Hamilton Depression Rating Scale-17 > 16) before and after 14-sessions of CBT monotherapy. Treatment outcome was assessed using the Beck Depression Inventory (BDI). Rostral anterior cingulate (rACC), anterior subcallosal cingulate (aSCC), and Brodmann’s area 25 (BA25) masks were used as seeds in connectivity analyses that assessed baseline rsFC and symptom severity, changes in connectivity related to symptom improvement after CBT, and prediction of treatment outcomes using whole-brain baseline connectivity.

**Results.:**

Pretreatment BDI negatively correlated with pretreatment rACC ~ dorsolateral prefrontal cortex and aSCC ~ lateral prefrontal cortex rsFC. In a region-of-interest longitudinal analysis, rsFC between these regions increased post-treatment (*p* < 0.05_FDR_). In whole-brain analyses, BA25 ~ paracentral lobule and rACC ~ paracentral lobule connectivities decreased post-treatment. Whole-brain baseline rsFC with SCC did not predict clinical improvement.

**Conclusions.:**

rsFC features of rACC and aSCC, but not BA25, correlated inversely with baseline depression severity, and increased following CBT. Subdivisions of SCC involved in top-down emotion regulation may be more involved in cognitive interventions, while BA25 may be more informative for interventions targeting bottom-up processing. Results emphasize the importance of subdividing the SCC in connectivity analyses.

## Introduction

Cognitive behavioral therapy (CBT) is an evidence-based treatment for major depressive disorder (MDD), yielding remission rates of 43–52% [1], which means about half of patients are ineffectively treated or left with significant residual symptoms. Understanding the neural mechanisms of CBT may allow development of treatment refinements to maximize its effectiveness. Identifying predictors of treatment outcome could advance the goal of personalized medicine, facilitating better matching of patients to likely effective treatments.

Aberrant activity of the subcallosal cingulate cortex (SCC) is a common theme across treatment efficacy prediction studies [2]. Using task-based fMRI, some groups have shown that eventual responders to CBT have low sustained reactivity to emotionally negative stimuli in SCC [3,4], while others have noted elevated SCC activity that normalizes with treatment [5,6]. Studies in depressive disorders have generally found hyperconnectivity between SCC and cortical regions as well as the default mode network [7]. Differences in the functional connectivity of the SCC that are predictive of treatment response have also been identified [2]. However, these studies used either pharmacological (e.g., selective serotonin reuptake inhibitors) [8] or neuromodulatory (e.g., transcranial magnetic stimulation) [9–12] treatment interventions; there is no consensus about which regions predict response to psychotherapy. Furthermore, the SCC includes two subregions that have significant cytoarchitechtonic differences and very little overlap in coactivation patterns [13]. Posterior SCC, identified as Brodmann’s area 25 (BA25), is involved in the bottom-up processing of emotion, while the anterior subregion (aSCC) is likely more involved in top-down control of emotions [13]. Conflicting results in the literature with regard to SCC involvement in treatment outcomes may be related to conflation of these two subregions falling under the broader “subgenual cingulate cortex” label. The cognitive, top-down emotion regulation strategies employed during CBT may recruit aSCC, while BA25 could be more responsive to pharmacological interventions.

The rostral anterior cingulate cortex (rACC), located at the genu of the corpus callosum, may also have features predictive of treatment outcome in depression. Early studies of regional glucose metabolism using positron emission tomography (PET) identified greater rACC glucose metabolism as a predictor of response to pharmacotherapy in depression [6,14,15], and rACC volume predicted response to an internet-based CBT intervention, while SCC volume did not [16]. A recent longitudinal study found that resting-state functional connectivity (rsFC) of the rACC with the salience network predicted reductions in depressive symptoms in general, irrespective of treatment type [17].

There has been growing interest in using rsFC as a tool to understand pathophysiology of MDD [7], and to identify predictors of treatment response [18,19]. In treatment-naïve participants with MDD, SCC rsFC differentially predicted the success of medication or CBT [19], highlighting a neural heterogeneity in the SCC in depression. Importantly, the seed used in that study was located in BA25, a subregion of SCC that likely plays a different role in the pathophysiology of depression from more anterior subregions. Parsing the SCC by its structural and functional subregions may be critical to characterizing the predictive capacity of the SCC. In addition, a longitudinal comparison of rsFC pre- and post-CBT could identify changes in neural circuitry that accompany treatment response, which will allow researchers to better understand the mechanism underlying CBT efficacy and to develop novel augmentation strategies based on this understanding.

Our group previously examined neurocognitive performance [20] and neural correlates of emotion regulation [21,22] in relation to CBT outcome. In the current study, using resting state fMRI (rsfMRI) data from the same set of subjects, we used the rACC, and two subregions of the SCC (BA25 and aSCC) as seeds and examined (a) correlates of depression symptom severity in MDD at baseline; (b) correlations between longitudinal changes in rsFC and clinical improvement; and (c) prediction of treatment outcome with CBT using baseline rsFC. A small sample of healthy volunteers (HVs) were also scanned at two timepoints and exploratory group contrasts are presented. To identify rsFC that is related to both baseline depression symptom severity and clinical improvement following CBT, we conducted longitudinal region-of-interest (ROI) analyses using results from analysis of baseline correlations with BDI in the MDD group. We anticipated that anterior cingulate rsFC with frontoparietal regions would be negatively correlated with depression severity at baseline, would predict CBT outcome, and would increase as a function of improvement following CBT.

## Methods and Materials

### Sample

Subjects gave written informed consent, as required by the Institutional Review Board of the New York State Psychiatric Institute (NYSPI). Participants were recruited with online and print advertisements as well as clinical referrals. Participants in the MDD group were diagnosed with MDD as assessed using the Structured Clinical Interview for DSM-IV (SCID) [23] and had a 17-item Hamilton Depression Rating Scale (HDRS) ≥16 [24]. HVs had no DSM-IV Axis 1 diagnoses. This sample has been previously described [21], and full inclusion and exclusion criteria are listed in Supporting Information (SI) 1.

### Clinical procedures and treatment

Depression severity was measured using the Beck Depression Inventory (BDI) [25]. After baseline magnetic resonance imaging (MRI) scanning, 14 45-min sessions of CBT for depression were administered over approximately 12 weeks according to a treatment manual [26]. Sessions occurred as close as possible to twice-weekly for 2 weeks and weekly thereafter. Both study therapists were MD- or PhD-level with extensive training in CBT, and met weekly for peer supervision. Sessions were audiotaped and at least one session per patient was assessed for adherence at the Beck Institute using the Cognitive Therapy Rating Scale [27]. BDI was assessed at every treatment session. A post-treatment rsfMRI scan was performed and BDI assessed at the conclusion of CBT.

Fifty-three participants (20 HVs and 33 patients with MDD) enrolled and completed baseline rsfMRI. Thirty-one of the 33 patients with MDD had usable scans, and one patient with MDD was excluded after baseline scanning due to subsequent detection of a diagnosis of current ETOH dependence. Time 2 scans were obtained for 10 HVs (without intervention) and 17 patients with MDD who completed CBT. One HV time 2 scan was excluded due to technical issues, and two patients with MDD did not have usable time 2 data. Nine patients with MDD did not complete 14 weeks of CBT monotherapy: five participants stopped treatment early, and antidepressant medication was added to CBT for four participants (two prior to and two after time 2 scanning) during the course of treatment due to clinical worsening. For the subjects who did not complete all 14 sessions of CBT monotherapy, a last observation carried forward analysis was applied, using the last BDI measurement before treatment was ended or antidepressant medication was started. See SI Table 1 for demographics and SI Figure 2 for study design and recruitment workflow.

### fMRI data acquisition

MRI scans were acquired on two 3T SignaHDx scanners using the same 8-channel head coil (General Electric Medical Systems, Milwaukee, WI). All but 16 scans (6 MDD and 1 HV pre and post, 2 HV pre) were acquired at NYSPI. The remaining were performed at Weill Cornell Medical College due to an upgrade to the NYSPI scanner. Main analyses did not correct for scan site since site and scanner manufacturer have been found to not significantly impact general linear model (GLM)-based rsFC [28]. To confirm this, all GLM analyses were repeated with scan site as a covariate. In general, the same regions/clusters appeared in both the main analyses and these control analyses. T1-weighted MRI scans were acquired using the following parameters: repetition time (TR) = ~6 ms, echo time (TE) = minimum 2,400 ms, flip angle = 8°, field-of-view (FOV) = 25.6 cm × 25.6 cm, slice thickness = 1 mm, number of slices = 178, matrix size = 256 × 256 pixels. Echo planar imaging (EPI) acquisition for rsfMRI was obtained using the following parameters: TR = 2,000 ms, TE = 28 ms, flip angle = 90°, FOV = 22.4 cm × 22.4 cm, slice thickness = 3.2 mm, spacing = 3.1 mm, 39 slices, matrix size = 64 × 64 pixels, 180 volumes.

### fMRI analysis

#### Preprocessing

Functional data were preprocessed and processed in Statistical Parametric Mapping version 8 (SPM8) [29,30]. Realigned T2*-weighted volumes were slice-time corrected, spatially transformed to the EPI Montreal Neurological Institute (MNI) template (12 degrees of freedom (DOF)), resampled to a standardized brain (MNI, 2 mm × 2 mm × 2 mm resolution), and smoothed with an 8-mm full-width half-maximum Gaussian kernel.

#### First-level analysis

The BA25 seed was defined using the BA25 mask available in WFU_Pickatlas [31,32]. aSCC and rACC seeds were defined by 8 mm spheres centered at [0 36 −6] and [0 38 6], respectively (SI Figure 1). Mean activity was extracted from these ROIs and used as regressors-of-interest in separate models. Nuisance regressors included six motion parameters, white matter, and cerebrospinal fluid signal, as well as spike confound regressors from fsl_motion_outliers [33] (default parameters and dvars) to adjust for volumes corrupted by large motion. In addition, a temporal high-pass filter (*f* > 1/128 Hz) was applied. For baseline data, beta-estimates for regressors-of-interest were passed to subsequent second level analyses. For subjects with longitudinal data, additional models which included both pre- and post-CBT sessions were estimated, and the contrast pre > post as well as the prebeta-estimates (for baseline prediction of treatment outcome) were passed to subsequent second level models or used for statistical learning analyses.

#### Second-level analysis

*Pretreatment rsFC:* A multiple regression model was estimated for each of the seeds in the MDD group (*n* = 30) with intercept, BDI, age, and sex as regressors to examine associations between rsFC with each seed and symptom severity in MDD at baseline. Baseline differences in rsFC between HVs and patients with MDD were also assessed using a one-way analysis-of-covariance (ANCOVA) with age and sex as nuisance covariates.

#### Longitudinal changes

Functional ROIs were generated from the peak coordinates of the baseline regression model and used for post hoc analyses to determine whether the rsFC that correlated with symptom severity at baseline changed following CBT. For this, we report adjusted *p*-values (FDR-corrected) for the one-sample *t*-test of mean change following CBT. Significant results from this analysis were further tested for correlations with change in BDI. To identify how CBT-induced changes in rsFC related to changes in symptom improvement in the MDD group, the pre > post contrast estimates in rsFC for each of the seeds was used as the outcome variable in separate multiple regression models, with intercept and percent change in BDI as regressors (note that in this model, negative *t*-values denote regions where decreases in rsFC correlate with symptom improvement). In addition, a one-sample *t*-test of pre- versus post-treatment contrast images in the MDD group (*n* = 19) was estimated to examine change in rsFC following treatment.

#### Prediction of treatment outcome

Baseline rsFC for each seed was used as the dependent variable in a regression model, with intercept and percent change in BDI from pre- to post-treatment used as regressors. Prediction of treatment outcomes was also tested using statistical learning analyses. Whole-brain rsFC beta maps from each seed were used as input features in statistical learning analyses to predict treatment outcome within the MDD group using leave-one-out cross validation. For each training set (*n* = 18), the top 1 through 10 features (voxels) were selected using Pearson correlation (with outcome variable) and used as predictors in a multiple linear regression model (using the *glmfit* function in Matlab). The predicted value of the left out sample was then estimated using the fitted model from the training set (using *glmval* function in Matlab). For each top *N* selected features, root-mean-squared deviation (RMSD), a measure of the differences between predicted values and actual values, was estimated and compared to a null distribution generated by shuffling improvement scores (relative to input brain scan labels) 200 times (2,000 total). We further tested whether positive results from the prediction analyses survived after adjustment for baseline symptom severity, age, and sex.

As a data reduction approach to identify candidate regions-of-interest for longitudinal analyses, results from baseline models correlating rsFC with BDI are reported with a family-wise error (FWE) cluster-extent corrected *p* < 0.1 threshold. All other GLM analyses are reported with a FWE cluster-extent *p* < 0.05. Correction for multiple comparisons was conducted using 3dClustSim (compiled December 11, 2018) with the -acf option (input parameters estimated using residuals from the SPM baseline regression with aSCC seed), and a cluster determining threshold of *p* < 0.001 for all models. The minimum cluster size for *N* = 2 and 2-sided testing was *k* = 163. This approach was based on recent recommendations to reduce false-positive rates when using cluster-extent correction [34,35] and includes a more accurate estimate of the noise smoothness values using a mixed model (Guassian plus a monoexponential) [36]. Statistical maps are displayed using xjview (http://www.alivelearn.net/xjview8/). Figures are displayed in neurological convention (i.e., right = right and left = left). All reported *p*-values are two-tailed unless otherwise indicated. For all GLM analyses, we also explored a whole-brain voxel-wise correction for multiple comparisons using the *randomize* function in FMRIB Software Library (FSL) with nonparametric threshold free cluster correction and 1,000 permutations [37] at *p* < 0.05, since this correction is more stringent and less sensitive to weaker, spatially distributed signals. Results that survived this additional whole-brain voxel-wise correction are marked in the corresponding tables with an asterisk.

## Results

### Pretreatment rsFC

**Figure 1.**
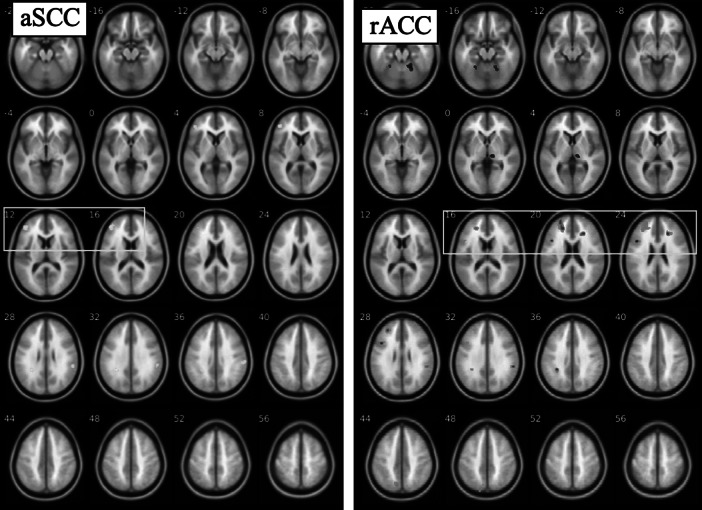
Anterior subcallosal cingulate (aSCC) and rostral anterior cingulate (rACC) resting-state functional connectivity associated with depression symptom severity (Beck Depression Inventory [BDI]) in major depressive disorder group at baseline (*N* = 30). For aSCC, regions negatively correlated with BDI include lateral prefrontal cortex (box), supramarginal gyrus, and parietal lobe; for rACC, regions positively correlated with BDI include fusiform and thalamus (top two rows), while regions negatively correlated with BDI include inferior frontal gyrus, dorsolateral prefrontal cortex (box), parietal cortex, and precuneus. Maps thresholded at *p* < 0.001, *k* > 40 for display purposes.

**Figure 2.**
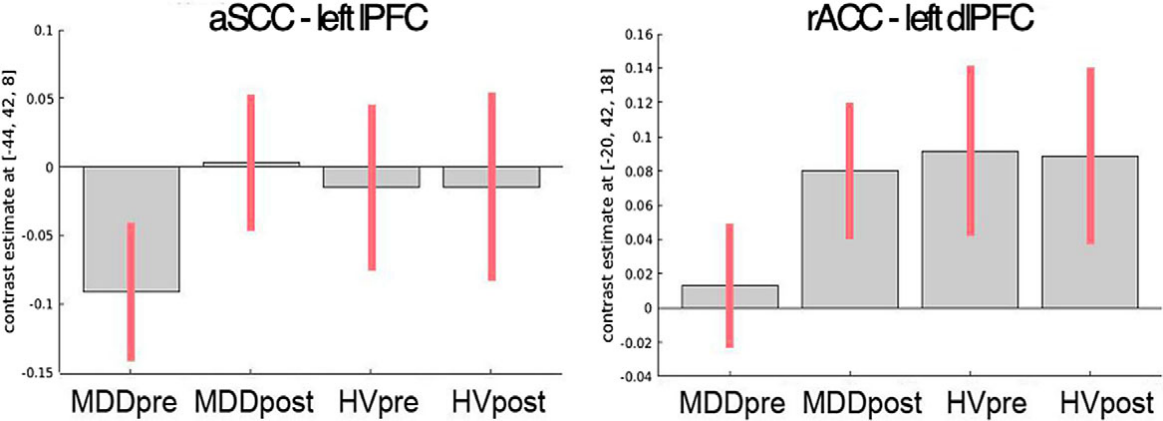
Anterior subcallosal cingulate (aSCC) ~ prefrontal cortical functional connectivity normalizes following cognitive behavioral therapy (CBT). Plots of average contrast estimates and 90% confidence intervals for major depressive disorder (MDD) and healthy volunteer pre- and post-scans for rostral anterior cingulate-dorsolateral prefrontal cortex and aSCC-lateral prefrontal cortex resting-state functional connectivity (rsFC). These rsFC correlated with depression symptom severity within MDD at baseline (top half of Table 1) and also changed following CBT (longitudinal ROI analysis *p* < 0.05 corrected, see “Results” section).

We used a relaxed threshold (*p* < 0.1_FWE_) to identify rsFC that correlated with baseline BDI as a data reduction approach to identify candidate ROIs for longitudinal analyses (Table 1). Baseline rsFC of the aSCC seed with a cluster in left lateral prefrontal cortex (lPFC) correlated negatively with baseline BDI (*p* < 0.1_FWE_, Figure 1), indicating that those patients who had greater connectivity between aSCC and left lPFC had lower symptom severity. BDI also correlated negatively with rsFC of the rACC seed with left dorsolateral prefrontal cortex (dlPFC) and correlated positively with a cluster in the cerebellum (*p* < 0.1_FWE_, Figure 2). No correlations between rsFC for the BA25 seed and BDI reached significance, and group-level comparisons of rsFC in HV with MDD at baseline yielded no significant results.

**Table 1.** rsFC correlations with BDI in MDD at baseline (top half; *p* < 0.001 CDT, *p* < 0.1 FWE cluster-extent corrected) and changes following 8-weeks CBT therapy (bottom half; *p* < 0.001 CDT, *p* < 0.05 FWE cluster-extent corrected)

Baseline correlation with BDI in MDD	*x*	*y*	*z*	Cluster size	*t*-value
rsFC with aSCC					
Frontal_Inf_Tri_L (left lPFC)	−44	42	8	158	*t*(29) = −4.515
rsFC with rACC					
Cerebelum_6_R	22	−56	−22	222	*t*(29) = 4.4831^a^
Frontal_Inf_Tri_L	−44	14	26	134	*t*(29) = −5.1965
Medial_Frontal_Gyrus (left dlPFC)	−20	42	18	358	*t*(29) = −4.6662^a^
rsFC with BA25					
No suprathreshold clusters					
Change following CBT in MDD	*x*	*y*	*z*	Cluster size	*t*-value
rsFC with aSCC					
Pre > post					
Precuneus_L	−14	−58	58	154^(n.s.)^	*t*(18) = 6.9581^a^
Percent reduction in BDI (post–pre/pre)					
No suprathreshold clusters					
rsFC with rACC					
Pre > post					
Paracentral_Lobule_L	−14	−32	66	203	*t*(18) = 5.7033^a^
Percent reduction in BDI					
No suprathreshold results					
rsFC with BA25					
Pre > post					
Paracentral_Lobule_R	4	−38	70	171	*t*(18) = 5.8974
Pre > post ~ change in BDI					
No suprathreshold results					

A looser *p* < 0.1 FWE cluster-extent corrected threshold was used for baseline analyses as a data reduction approach to identify candidate ROIs for longitudinal analyses. For pre > post comparisons, positive (negative) *t*-values denote group averaged reductions (increases) in FC following treatment. For pre > post versus change in BDI, negative *t*-values denote regions which exhibit greater rsFC decreases with greater improvement, whereas positive *t*-values denote regions which exhibit greater rsFC increases (or less decreases) with greater improvement. Change in BDI is quantified as percent reduction in BDI (post–pre/pre). n.s.: cluster-extent did not survive correction.

Abbreviations: aSCC, anterior subcallosal cingulate; BA25, Brodmann’s area 25; BDI, Beck Depression Inventory; CBT, cognitive behavioral therapy; CDT, cluster determining threshold; dlPFC, left dorsolateral prefrontal cortex; lPFC, left lateral prefrontal cortex; MDD, major depressive disorder; rACC, rostral anterior cingulate; rsFC, Resting-state functional connectivity.

a Cluster in which one or more voxels reached *p* < 0.05 corrected using whole-brain voxel-wise correction for multiple comparisons (see “Methods” section).

### Longitudinal analyses

ROI analyses were conducted to examine whether rsFC that was correlated with depression symptom severity at baseline changed following treatment in the MDD group. Of the four coordinates listed in Table 1 (top half), aSCC-left lPFC (*t*_(18)_ = 3.29, *p* = 0.016_FDR_), and rACC-left dlPFC rsFC (*t*_(18)_ = 2.62, *p* = 0.034_FDR_) increased following treatment. The aSCC-left lPFC increased from negative rsFC pretreatment to near zero following treatment, and this change in rsFC was positively correlated with change in BDI at a trend level (*t*_(18)_ = 2.04, *p* = 0.056_FDR_). Connectivity between rACC-left dlPFC also normalized from near zero to positive values post-treatment (Figure 2). Change in rACC-left dlPFC rsFC did not show evidence for correlation with change in symptom improvement (*t*_(18)_ = 1.45, *p* = 0.17).

**Figure 3.**
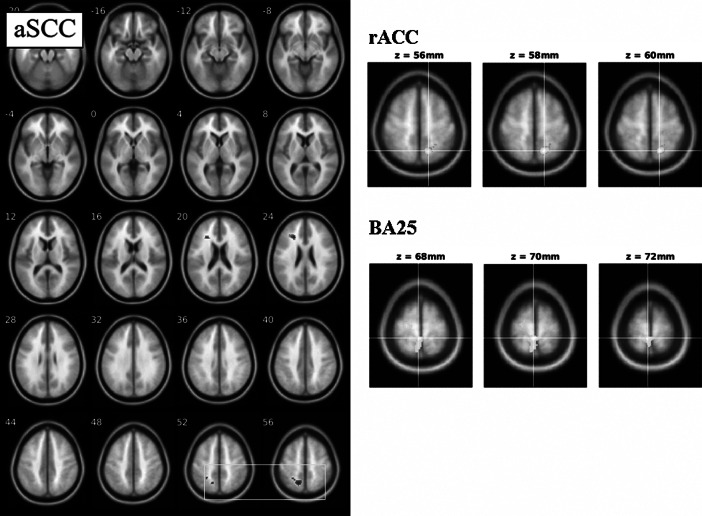
Anterior subcallosal cingulate (aSCC), rostral anterior cingulate (rACC), and BA25 resting-state functional connectivity pre- versus post-differences following cognitive behavioral therapy in major depressive disorder (*N* = 19 participants). For aSCC, regions that decreased include precuneus (box); for rACC and BA25, regions that decreased include paracentral lobule (right panels). For display purposes, maps are thresholded at *p* < 0.001, *k* > 40.

In a whole-brain analysis, we identified rsFC that changed following 8-weeks CBT in MDD (*p* < 0.05, corrected, Table 1, Figure 3). rACC rsFC with paracentral lobule (PCL) decreased following treatment. BA25-PCL rsFC also decreased (Table 1). Group-averaged responses (pre- and post-treatment in MDD, pre- and post-no intervention in HV) for these regions showing significant change in rsFC following treatment (Table 1), were inspected to see which responses appeared to normalize following treatment. In general, pre–post-treatment changes tended to “normalize,” or change in a direction towards that observed in both HV pre and HV post no intervention scans (SI Figure 3).

**Figure 4.**
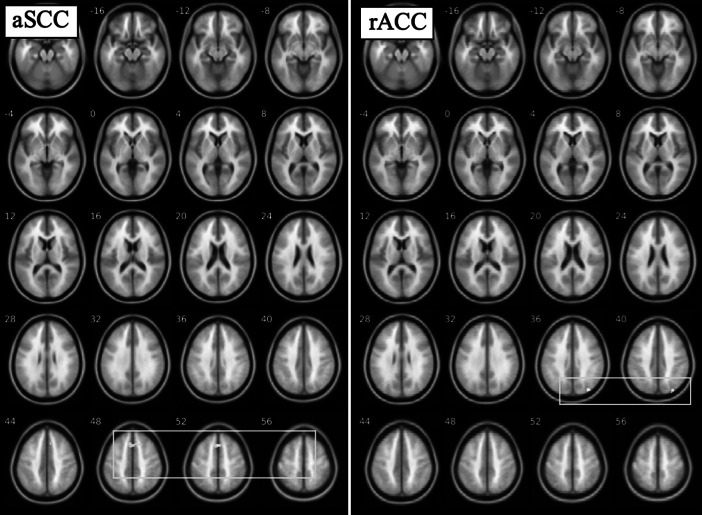
Anterior subcallosal cingulate (aSCC) correlations with improvement following cognitive behavioral therapy in major depressive disorder (*N* = 19 participants). For aSCC, regions with greater resting-state functional connectivity decreases correlating with greater improvement included dorsal ACC/SMA (box). For display purposes, maps are thresholded at *p* < 0.001, *k* > 40.

To identify changes in rsFC following CBT that were associated with clinical improvement, we correlated pre–post-treatment changes in rsFC with percentage change in BDI. No clusters survived correction at *p* < 0.05. A table of clusters surviving a relaxed threshold (*p* < 0.1_FDR_) can be found in SI Table 3, and shows clinical improvement negatively correlated with rsFC between aSCC and the dorsal anterior cingulate/supplementary motor area (dACC/SMA) (Figure 4).

### Predicting treatment outcome

Whole-brain baseline BA25 and rACC rsFC maps did not predict treatment outcome (all *p* > 0.5). Baseline rsFC of aSCC predicted treatment outcome at the trend level with higher than chance performance (RMSD) when selecting the top five through seven features (voxels). The average performance (variance explained) across the prediction tasks when using the top 1 through 10 features was 23% (mean RMSD *p* = 0.07). Informative voxels in this prediction included frontal operculum and temporal cortex. However, these rsFC maps did not predict CBT outcome when features were first adjusted for baseline symptom severity (*p*-values >0.5), indicating that the aSCC rsFC findings above likely reflect associations with baseline symptom severity.

## Discussion

We used three seeds in the ACC to assess rsFC before and after a CBT intervention in patients with MDD. In general, results suggested that the strength of rsFC between the aSCC and rACC seeds and cortical areas involved in top-down cognitive control of emotions tracked symptom severity. Additionally, connectivity between parietal areas and all three seeds was reduced following a course of CBT, increasing their similarity with those seen in healthy volunteers and suggesting a normalization of network functioning following treatment.

### Pretreatment rsFC

Pretreatment depression symptom severity was negatively correlated with connectivity between aSCC and left lPFC and between rACC and dlPFC, such that more severely depressed individuals had lower rsFC between these regions. Moreover, the strength of rsFC increased between these regions following CBT, and increased aSCC-lPFC rsFC showed trend level evidence for correlation with symptom severity improvement. The lPFC is recruited during voluntary behavioral and cognitive control of emotions [38], techniques that are taught during CBT. A possible explanation of increased aSCC-lPFC rsFC is that effective CBT treatment may enhance signaling from the lPFC to the aSCC, strengthening the bias lPFC can exert on evaluation of emotion in this region [13] thus improving emotion-regulation capacity. However, it is important to note that the nature of the rsFC analyses performed here address neither the directionality of effects, nor causality. The rACC is an integration region with extensive connections throughout cortical and subcortical areas [39] and is theorized to play a key regulatory role between the dorsal (attention and cognition) and ventral (vegetative and somatic) compartments in the limbic–cortical dysregulation model of depression [14]. Increased rsFC between rACC and dlPFC could indicate improved coordination between rACC and this cognition system. Importantly, no significant rsFC with the BA25 seed was identified at baseline. This is congruent with reported functional differences between anterior and posterior SCC, whereby CBT is more likely to modulate the top-down processing of emotion that occurs in aSCC rather than the bottom-up emotion processing that occurs with BA25 [13]. It is therefore imperative to recognize the subdivisions of the SCC in future neuroimaging studies to maximize the reproducibility of results.

### Longitudinal changes in rsFC following CBT

Following CBT, there was decreased connectivity in both rACC and BA25 with clusters in the PCL, resulting in a more normalized pattern of activity. The PCL, along with the SCC, is a densely connected part of the “structural core” of the cortex involved in functional integration of cognition and motor response [40]. Together, BA25 and PCL are involved in emotional modulation of pain perception [41], and integrating interoceptive awareness with self-referential stimuli [42], which may relate to somatosensory symptoms and negativity biases common in depression. Decreased connectivity between rACC and the more anterior part of PCL observed in this study is more likely due to cognitive changes from CBT. Both rACC and PCL are part of an emotion processing network whose activity decreases during cognitive reappraisal [43], a skill learned with CBT. Emotion-induced activity in this more anterior part of PCL is correlated with the amount of emotional (versus cognitive) words participants used to describe themselves, suggesting this region is recruited during embodied, affective emotion processing [44]. A reduction in hyperconnectivity between rACC and PCL might be expected with improved cognitive control of emotions, such as reduced rumination and negative self-referential processing. Importantly, both seeds showed a normalization of connectivity with distinct clusters in the parietal cortex. This spatial diffusion of rsFC targets would reduce the power to find an effect in studies that do not subdivide the SCC. Future research combining rsFC changes in parietal-cingulate connectivity with comprehensive measures of depressive symptoms may help build a more complete understanding of the contributions these areas have to the disorder.

### Limitations

Pre–post changes in rsFC in the MDD group are not necessarily a result of treatment. To address these limitations and identify regions whose changes were more likely to be due to treatment intervention, we inspected pre and post-treatment responses in patients with MDD, and estimated models which identified regions whose changes correlated with measures of depression symptom severity. Given the relatively small longitudinal sample size (*n* = 19), longitudinal and treatment outcome predictions should be interpreted with caution. Future studies with larger sample sizes are required to determine whether these findings generalize to independent cohorts. In future work, it will be important to compare results when using a clinician-administered scale versus a self-report outcome measure. Note that earlier work by our group found significantly stronger and more widespread correlations between a related brain imaging measure, resting glucose metabolism assessed by FDG-PET, and the outcome measure used in this study, the BDI, than was found with a clinician-administered scale, the HDRS [45]. One benefit of using the BDI is that it assesses the cognitive aspects of depression in greater depth than the HDRS, which focuses more on neurovegetative symptoms. In addition, we had more frequent clinical assessments in this study with the BDI (at each treatment session) than with the HDRS (every four sessions), allowing us to use more recent depression severity measure for participants who did not complete 14 sessions of CBT monotherapy with last observation carried forward. The rate of participant noncompletion may be partially attributed to baseline depression severity of the sample. Finally, in the absence of randomization to different treatment modalities, including a placebo condition, it is not possible to attribute observed rsFC changes to CBT specifically.

## Data Availability

The data that support the findings of this study will be available in a deidentified form upon reasonable request from the authors.

## Clinical Trial Registration Information.

Study Title: Predictors of Treatment Outcome With Cognitive Behavioral Therapy for Depression, ClinicalTrials.gov URL: https://clinicaltrials.gov/ct2/show/NCT01922219?term=NCT01922219, Identifier: NCT01922219.

## Supplementary material

For supplementary material accompanying this paper visit http://dx.doi.org/10.1192/j.eurpsy.2020.34. click here to view supplementary material
