# Depression-related anterior cingulate prefrontal resting state connectivity normalizes following cognitive behavioral therapy - CORRIGENDUM

**DOI:** 10.1192/j.eurpsy.2020.61

**Published:** 2020-07-17

**Authors:** Spiro P. Pantazatos, Ashley Yttredahl, Harry Rubin-Falcone, Ronit Kishon, Maria A. Oquendo, J. John Mann, Jeffrey M. Miller

**Affiliations:** 1 Molecular Imaging and Neuropathology, New York State Psychiatric Institute, New York, New York, USA; 2 Department of Psychiatry, Columbia University Irving Medical Center, New York, New York, USA; 3 Department of Electrical Engineering and Computer Science, University of Michigan, Ann Arbor, Michigan, USA; 4 Department of Psychiatry, Perelman School of Medicine, University of Pennsylvania, Philadelphia, Pennsylvania, USA

In the aforementioned article, the incorrect corresponding author was published; this has since been updated. The authors apologize for this error.
