# Supplementary material for: Depression-related anterior cingulate prefrontal resting state connectivity normalizes following cognitive behavioral therapy
Source: Eur Psychiatry. 2020 Apr 14;63(1):e37. doi: 10.1192/j.eurpsy.2020.34 (PMC7355178; doi:10.1192/j.eurpsy.2020.34)

Supplementary Material for:
Rostral and subgenual cingulate resting state functional connectivity in major depressive disorder: predictors and correlates of cognitive behavioral therapy outcome

Authors: Spiro P. Pantazatos, Ph.D.^1,2^, Ashley Yttredahl, Ph.D.^1,2^, Harry Rubin-Falcone, B.A.^2,3^, Ronit Kishon, Ph.D.^2^, Maria A. Oquendo, M.D., Ph.D.^4^, J. John Mann, M.D.^1,2^, Jeffrey M. Miller, M.D.^1,2,*^

^1^Molecular Imaging and Neuropathology, New York State Psychiatric Institute, New York, NY; ^2^Department of Psychiatry, Columbia University Irving Medical Center, New York, NY; ^3^Department of Electrical Engineering and Computer Science, University of Michigan, Ann Arbor, MI.; ^4^Department of Psychiatry, Perelman School of Medicine, University of Pennsylvania, Philadelphia, PA, USA

* To whom correspondence should be addressed:

E-mail: jm2233@cumc.columbia.edu

Contents:

Supplementary Tables 1-3

Supplementary Figures 1-3 Legends
Supplementary Figures 1-3

Running title: Subgenual cingulate functional connectivity following CBT in MDD

**Supplemental Information 1.** Inclusion and Exclusion Criteria

MDD inclusion criteria included: 1) Age 18-60; 2) A DSM-IV diagnosis of Major Depressive Disorder, assessed using the Structured Clinical Interview (SCID) for DSM-IV (First et al. 1995) 3) 17-item HRSD score ≥16 (M. Hamilton 1960); 4) lack of significant benefit from any current psychiatric medications (if applicable); 5) ability to tolerate three-week medication washout from current psychiatric medications (1 participant was taking antidepressant medication at enrollment and underwent washout prior to neuroimaging and treatment); 6) Capacity to provide informed consent. Exclusion criteria for MDD subjects included: 1) unstable medical conditions; 2) current alcohol or substance use disorder (past diagnosis allowed if in remission for ≥6 months); 3) other current or past major psychiatric disorders (comorbid anxiety disorders were not excluded). 4) pregnancy, currently lactating, planning to conceive during the course of study participation or abortion in the past two months; 5) dementia; 6) a neurological disease or prior head trauma with evidence of cognitive impairment; 7) a first-degree family history of schizophrenia if the subject is less than 33 years old (to exclude possible prodromal phase of schizophrenia); 8) currently taking fluoxetine (due to long half-life preventing biologically adequate washout within clinically appropriate duration); 9) contraindication to CBT as primary treatment for depression, including history of non-response to an adequate previous trial of CBT for depression of at least eight sessions, active psychotic symptoms, or severe suicidal ideation which includes a plan; 10) metal implants or paramagnetic objects contained within the body (including heart pacemaker, shrapnel, or surgical prostheses); 11) claustrophobia severe enough to interfere with MRI scanning; 12) weight that exceeds 350 lbs or inability to fit into MRI scanner. Healthy control inclusion criteria included: 1) Age 18-60; 2) absence of current or past DSM-IV Axis 1 diagnosis; 3) capacity to provide informed consent. Healthy control exclusion criteria include items 1,2,4,5,6,10,11, and 12 from the MDD exclusion criteria in addition to 1) a first-degree relative with history of major depression, schizophrenia, schizoaffective disorder, or suicide attempt; 2) two or more first degree relatives with a history of substance dependence.

**Supplementary Table 1.** Demographics Table

|  | HV | MDD | Statistic |
| --- | --- | --- | --- |
| *Baseline, Cross-Sectional Sample* | *N = 20* | *N = 30* |  |
| Age (Mean Years, Sd) | 32.4 (10.0) | 34.1 (10.0) | *t*(41) = -0.6, *P* = 0.55 |
| Gender (Number, % Female) | 12 (60%) | 19 (63%) | *Chi^2^* = 0.06,*P* = 0.81 |
| BDI Score (Mean, Sd) | 1.0 (2.6) | 28.4 (7.2) | *t*(39) = 19.0, *P* < 10E-10 |
| *Longitudinal Subsample* | *N* = 10 | *N* = 19 |  |
| Age (Mean Years, Sd) | 33.1 (7.7) | 32.6 (8.5) | *t*(20) = 0.17, *P* = 0.86 |
| Gender (Number, % Female) | 7 (70%) | 13 (68%) | *Chi^2^* = 0.009, *P* = 0.93 |
| Pre BDI (Mean, Sd) | 0 (0) | 29.7 (7.1) | *N/A* |
| Post BDI (Mean, Sd) | 0.1 (0.32) | 14.4 (8.0) | *N/A* |
| Pre-Post BDI | *N/A* | 15.3 (12.4) | *t*(18) = 5.4, *P* < 2.1E-5 |

**Supplementary Table 3. All clusters surviving p<0.001 CDT, p<0.1 FWE cluster-extent correction.** rsFC for all three seed regions correlation with BDI in MDD at baseline (top half) and changes following 8-weeks CBT therapy (bottom half). For *Pre>Post* comparisons, positive (negative) t-values denote group averaged reductions (increases) in FC following treatment. For pre>post vs. change in BDI, negative t-values denote regions which exhibit greater rsFC decreases with greater improvement, whereas positive t-values denote regions which exhibit greater rsFC increases (or less decreases) with greater improvement. Change in BDI is quantified as percent reduction in BDI (post – pre / pre)**.**

| **Baseline correlation with BDI in MDD** | ***x*** | | ***y*** | | ***z*** | **Cluster size** | ***t*-value** |
| --- | --- | --- | --- | --- | --- | --- | --- |
| **rsFC with aSCC** |  | |  | |  |  |  |
| Frontal_Inf_Tri_L (left lPFC) | -44 | | 42 | | 8 | 158 | *t*(29) = -4.515 |
| **rsFC with rACC** | | | | | | | |
| Cerebelum_6_R | 22 | | -56 | | -22 | 222 | *t*(29) = 4.4831 ^a^ |
| Frontal_Inf_Tri_L | -44 | | 14 | | 26 | 134 | *t*(29) = -5.1965 |
| Medial_Frontal_Gyrus (left dlPFC) | -20 | | 42 | | 18 | 358 | *t*(29) = -4.6662 ^a^ |
| **rsFC with BA25** |  | |  | |  |  |  |
| No suprathreshold clusters |  | |  | |  |  |  |
| **Change following CBT in MDD** | ***x*** | | ***y*** | | ***z*** | **Cluster size** | ***t*-value** |
| **rsFC with aSCC** | | | | | | | |
| *Pre > Post* | | | | | | |  |
| Precuneus_L | -14 | | -58 | | 58 | 154 | *t*(18) = 6.9581 ^a^ |
| *Percent reduction in BDI (post – pre / pre)* |  |  | |  | |  |  |
| Supp_Motor_Area_L | 0 | | 20 | | 52 | 133 | *t*(17) = -5.8012 |
| **rsFC with rACC** |  |  | |  | |  |  |
| *Pre > Post* | | | | | | |  |
| Caudate_L | -6 | | 20 | | 14 | 142 | *t*(18) = -5.3534 |
| Paracentral_Lobule_L | -14 | | -32 | | 66 | 203 | *t*(18) = 5.7033 ^a^ |
| *Percent reduction in BDI* |  | |  | |  |  |  |
| No suprathreshold results |  | |  | |  |  |  |
| **rsFC with BA25** |  | |  | |  |  |  |
| *Pre > Post* |  | |  | |  |  |  |
| Paracentral_Lobule_R | 4 | | -38 | | 70 | 171 | *t*(18) = 5.8974 ^a^ |
| *Pre > Post ~ change in BDI* |  | |  | |  |  |  |
| No suprathreshold results |  | |  | |  |  |  |

*^a^ indicates cluster in which one or more voxels reached p<0.05 corrected using whole-brain voxel-wise correction for multiple comparisons (see methods).*

**Supplementary Figure 1. Locations of seed regions.** Slices showing ROIs for Brodman Area 25 (BA25), subgenual anterior cingulate cortex (aSCC) and rostral anterior cingulate cortex (rACC).

**Supplementary Figure 2. Study design and recruitment workflow.**

**Supplementary Figure 3.** For regions that changed rsFC following treatment (main text Table 1, bottom half) we visualized and plotted group-averaged responses pre and post treatment in MDD and HV groups. aSCC-PCUN rsFC went from slightly negative to more negative (right panel), while for rACC rsFC with left caudate increased following treatment (i.e. from zero to positive) with post-treatment responses more similar to HV pre and post responses. Regions that decreased (i.e. paracentral lobule went from slightly negative to more negative in MDD and also appeared to normalize following treatment (i.e. resemble HV pre and post responses which were also negative, data not shown). Plots of average contrast estimates and 90% confidence intervals for MDD and HV pre and post scans. Regions are peak coordinates from select clusters in Figure 3 and Table 3 (rsFC showing longitudinal changes following CBT in MDD).

**Supplemental Figure 1.**

**
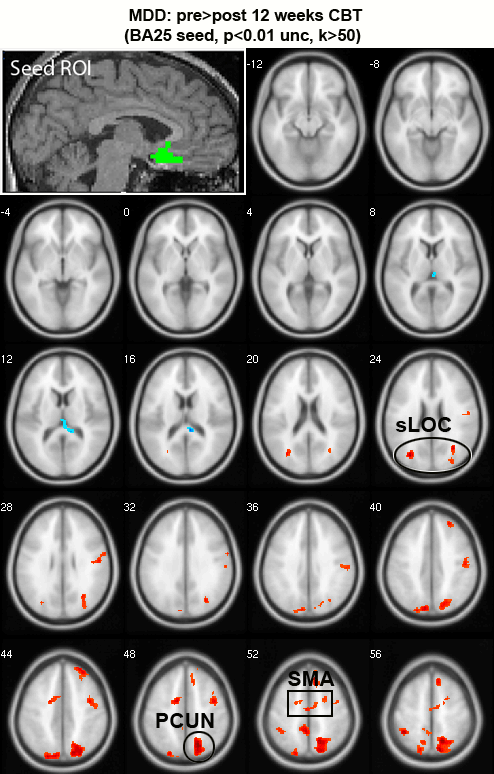

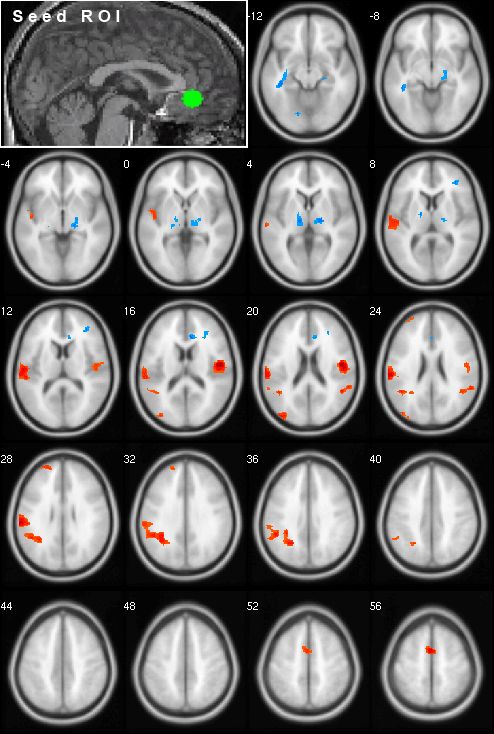
**

aSCC

BA25

rACC

**Supplementary Figure 2

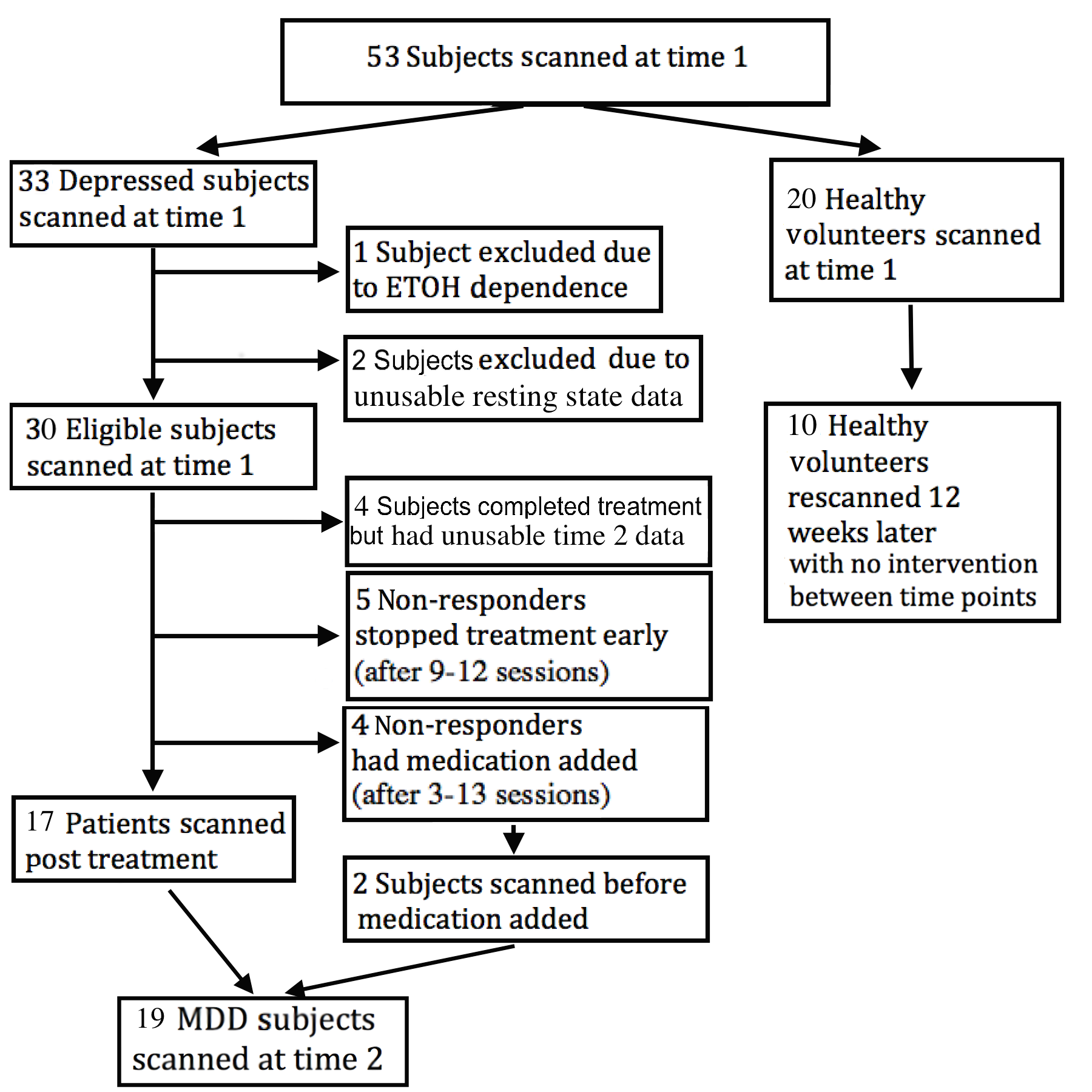
**

**Supplementary Figure 3.**

aSCC ~ precuneus

rACC ~ left caudate


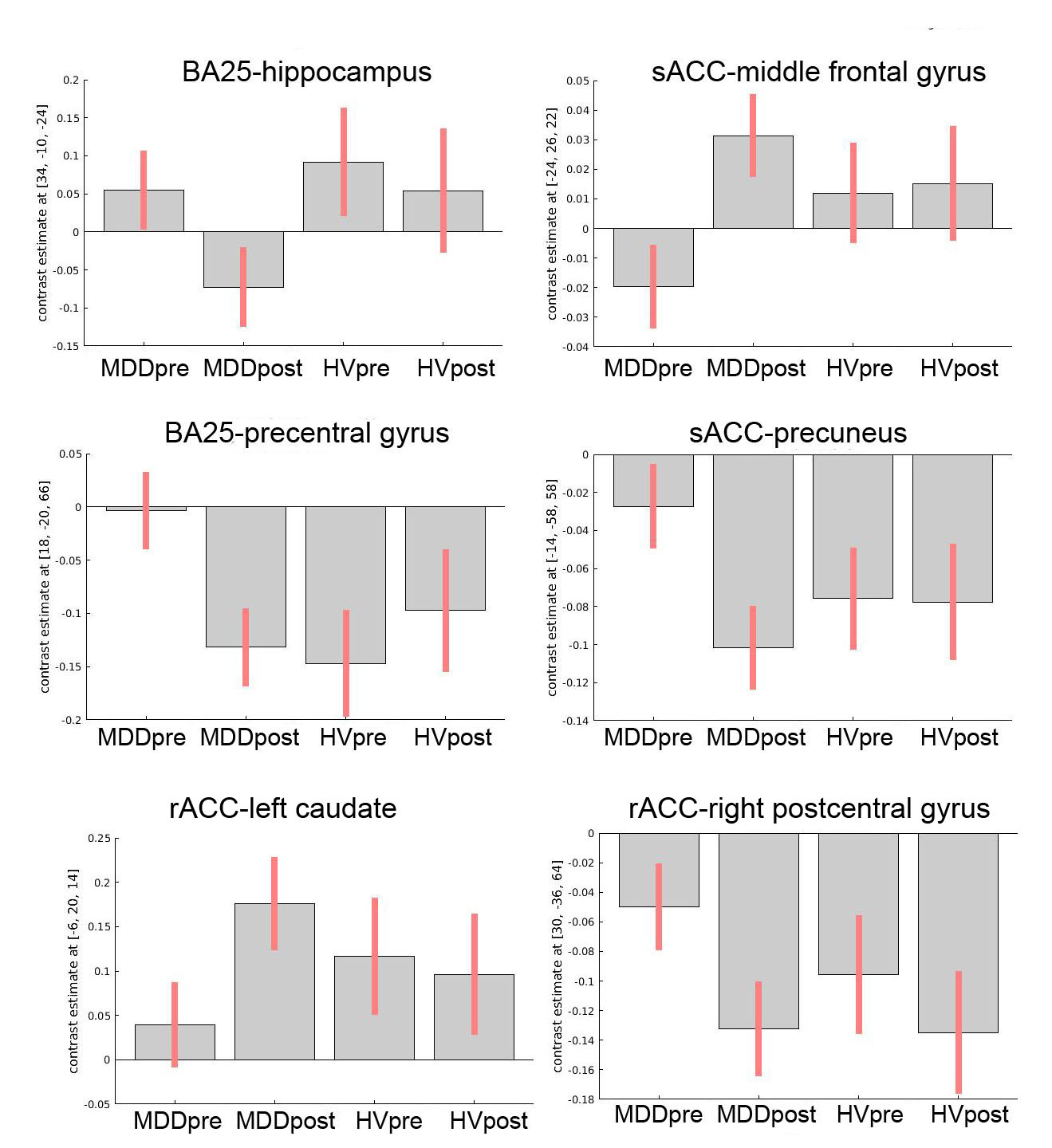

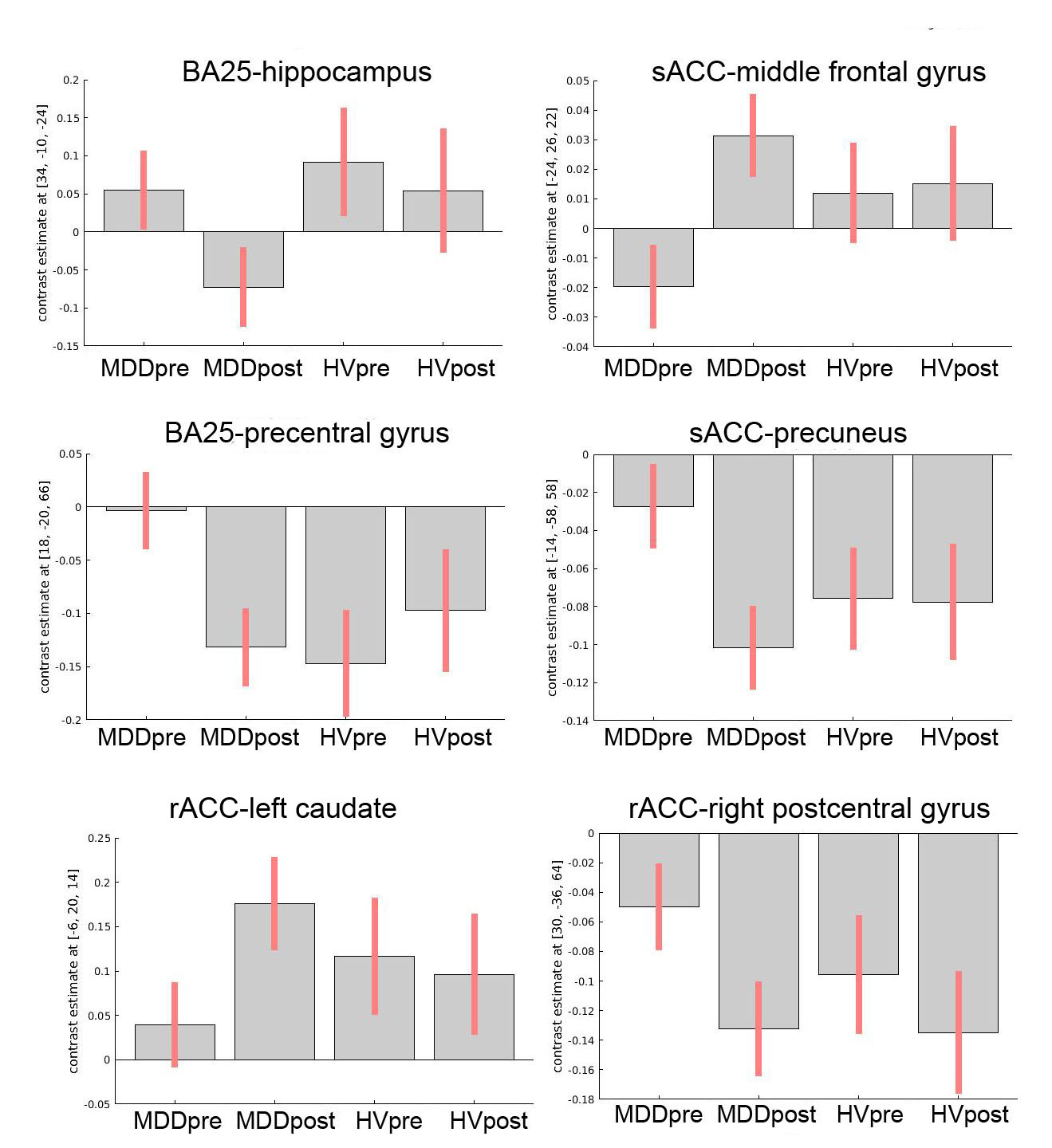

Supplement: Supplementary file 1 [file S0924933820000346sup001.docx]
